# Efficacy of Yijinjing combined with Tuina for patients with non-specific chronic neck pain: study protocol for a randomized controlled trial

**DOI:** 10.1186/s13063-021-05557-2

**Published:** 2021-09-03

**Authors:** Ziji Cheng, Ziying Chen, Fangfang Xie, Chong Guan, Yuanjia Gu, Ruiping Wang, Yanli You, Fei Yao

**Affiliations:** 1 School of Acupuncture-Moxibustion and Tuina, Shanghai University of Traditional Chinese Medicine, Shanghai, China; 2 Clinical Research Center, Shanghai Skin Diseases Hospital, Tongji University, Shanghai, China; 3 Department of Traditional Chinese Medicine, Changhai Hospital, Naval Medical University, Shanghai, China

**Keywords:** Non-specific chronic neck pain, Efficacy, Randomized controlled trial, Tuina, Yijinjing

## Abstract

**Background:**

Non-specific chronic neck pain (NCNP) is a common musculoskeletal disorder which has caused a huge economic burden due to its expensive health costs and high re-occurrence rate. Yijinjing and Tuina are widely used for non-specific chronic neck pain in China. But there is little scientific evidence to evaluate their efficacy for NCNP. The aim of this research is to compare the efficacy of Yijinjng combined with Tuina versus Tuina for patients with NCNP.

**Methods/design:**

A randomized controlled trial in which 102 patients with non-specific chronic neck pain will be recruited and randomly allocated to either the Tuina group or the Yijinjng combined with Tuina group in a 1:1 ratio. The interventions for both groups will be carried out three times a week for 8 weeks. The patients in the two groups will receive follow-up 1 month after the intervention. The primary outcome will be the changes in the visual analog scale (VAS). Secondary outcomes will be measured by the Neck Disability Index (NDI), Self-Rating Anxiety Scale (SAS), and Tissue Hardness and Active Range of Motion (AROM). The data will be analyzed at the baseline, 4 weeks during the intervention, at the end of the intervention, and 1 month after the intervention. The significance level sets as 5%. The safety of interventions will be evaluated after each treatment session.

**Discussion:**

The purpose of this trial is to determine whether Yijinjing combined with Tuina is not inferior to Tuina for patients with NCNP. This study will provide clinicians and stakeholders much-needed knowledge for a complementary and alternative therapy for patients with non-specific chronic neck pain.

**Trial registration:**

ChiCTR registry (ChiCTR) 2000036805. Registered on August 25, 2020

## Introduction

Non-specific chronic neck pain (NCNP) is a serious health and public problem worldwide. Neck pain appears between the occipital condyle and C7 in the neck region [1]. Neck pain can be divided into specific neck pain and non-specific neck pain. Non-specific neck pain is also known as mechanical neck pain which is defined as simple neck pain without specific pathological changes and neurological impairments; it can be diagnosed as non-specific chronic neck pain if the symptoms persist more than 3 months [2]. Two-thirds of the adult population suffer from non-specific chronic neck pain [3]. According to the research, women are more likely to be affected than men [4]. The annual incidence of NCNP is increasing because of sedentary lifestyle and working conditions [5, 6]. Patients’ quality of life and efficiency of work decrease due to chronic pain, and high treatment expenses also cause a huge burden to society [7].

The mechanism of NCNP is still not clear. The researchers explained the mechanism from different aspects, such as the mechanical factors, EMG, and ROM. Altered muscle cross-sectional area, thickness, size, and activity of deep neck muscles have been mentioned repeatedly in the previous [8–10]. Rahnama et al. [11] showed the change of altered EMG activity and the atrophy of deep neck extensor in patients with NCNP which is thought to be the recurrence of NCNP. Barnsley et al. [12] demonstrated that limited ROM aggravates the tightness of the muscles surrounding the neck and joint adhesion which also leads to a decrease in bio-mechanical function of the neck, and this condition causes non-specific chronic neck pain. In addition, much attention has been paid to the scapular region. Dyskinesia of the scapula and misalignment of the scapula always follow with NCNP [13, 14]. In addition, according to the “Bio-Psycho-social” framework, repetitive and sedentary working conditions and postural abnormalities also contribute to NCNP [15]. Anxiety and depression are also vital psycho-social factor which is thought to be associated with the existence of higher levels of pain in musculoskeletal pain conditions [16].

For most NCNP patients, first-line medication options always include analgesics like acetaminophen or non-steroidal anti-inflammatory drugs (NSAIDs), but the effects of these drugs vary from person to person and they often do harm to the digestive, blood, urinary, and other systems because of the long-term use [17–19]. So, various complementary treatments have become more and more popular. Exercise therapy, ultrasound, acupuncture, electrical nerve stimulation, and manual therapy have also been used widely in treating NCNP [20]. However, poor standardization of experiments, small sample size, low-quality control, and insufficient objective index caused controversy about their efficacy.

Tuina therapy is also called Chinese massage [21]. Tuina has also been proved to be a feasible way to treat neck pain and has been widely used in China [22]. As an important part of Chinese traditional medicine, Tuina is a manual therapy with anatomical and physiological principles, putting emphasis on meridians and acupoints [23]. Tuina therapy mainly includes two parts: soft tissue manipulation and spinal manipulation. Soft tissue manipulation techniques include stroking, kneading, and drumming, which are also found in some Western massage techniques, and spinal manipulation also combined with high-velocity low-amplitude thrust manipulation techniques [23]. Two systematic reviews had shown that Tuina therapy can reduce pain and muscle tension for patients with non-specific neck pain [24, 25]. Tuina therapy acts on soft tissue and connective tissue that may lead to local biochemical changes that regulate local blood circulation, improve muscle flexibility, enhance lymph movement, and loosen connective tissue adhesion, which may alternately improve local injury and inflammation of the reuptake mediator [26]. Chinese traditional exercise is also a kind of exercise therapy which puts attention on the coordination of posture, meditation, and breathing [27]. Exercise therapy has been proved to be good for non-specific chronic neck pain [28]. Yijinjing is an ancient Chinese traditional exercise which has been widely practiced for keeping fit and treating diseases. Yi means change, Jin refers to muscles and sinews, and Jing means methods, so Yijinjing means a series of exercises to change the muscle and sinews literally. Yijinjing is a low-intensity, non-competitive, and non-impact exercise. TCM doctors often apply Yijinjing as a complementary therapy to NCNP. Yijinjing can reduce neck pain and disability, as well as reduce stress, anxiety, and depression by unique movements [29–31]. Based on previous researches, people can improve sub-health, reduce pain, and promote immune cell by practicing Yijinjing regularly [32, 33]. However, to confirm these findings, more studies with larger sample sizes, standardized trials, and adverse event reports are needed.

Studies have shown that a single treatment plan is not effective, so the combination therapy has received more attention and is recommended by related scholars [34, 35]. There is quite a little evidence for the efficacy of Tuina on non-specific chronic neck pain, especially when combined with Yijinjing. We hypothesize that Tuina and Yijinjing have beneficial effects on non-specific chronic neck pain because that is a case with subacute and long-lasting neck pain [36]. We want to further explore whether Tuina combined with Yijinjing exercises can play a better role in pain, disability, and negative emotions. Thus, we designed a randomized controlled trial (RCT) to prove our hypothesis. This trial will provide a solid clinical foundation for the efficacy of Yijinjng combined with Tuina. It will be served as a prospective experiment as well.

## Objectives

This study is a randomized, evaluator- and statistician-blinded, parallel-controlled, superiority trial.

The purpose of this trial will be to assess the following:

1. Whether Tuina combined with Yijinjing is not inferior to Tuina regarding pain, disability, and negative emotions for patients with NCNP

### Specific primary objective

The specific primary objective is to determine the change in the visual analog scale (VAS) pain scores from baseline, 4 weeks during the intervention, at the end of the intervention, and 1 month after the intervention within and between the two groups.

### Specific secondary objectives

The specific secondary objectives are to determine the change in scores of Neck Disability Index (NDI), Self-Rating Anxiety Scale (SAS), and Tissue Hardness and Active Range of Motion (AROM) from baseline, 4 weeks during the intervention, at the end of the intervention, and 1 month after the intervention within and between the two groups.

## Methods/design

### Study design

**Fig. 1:**
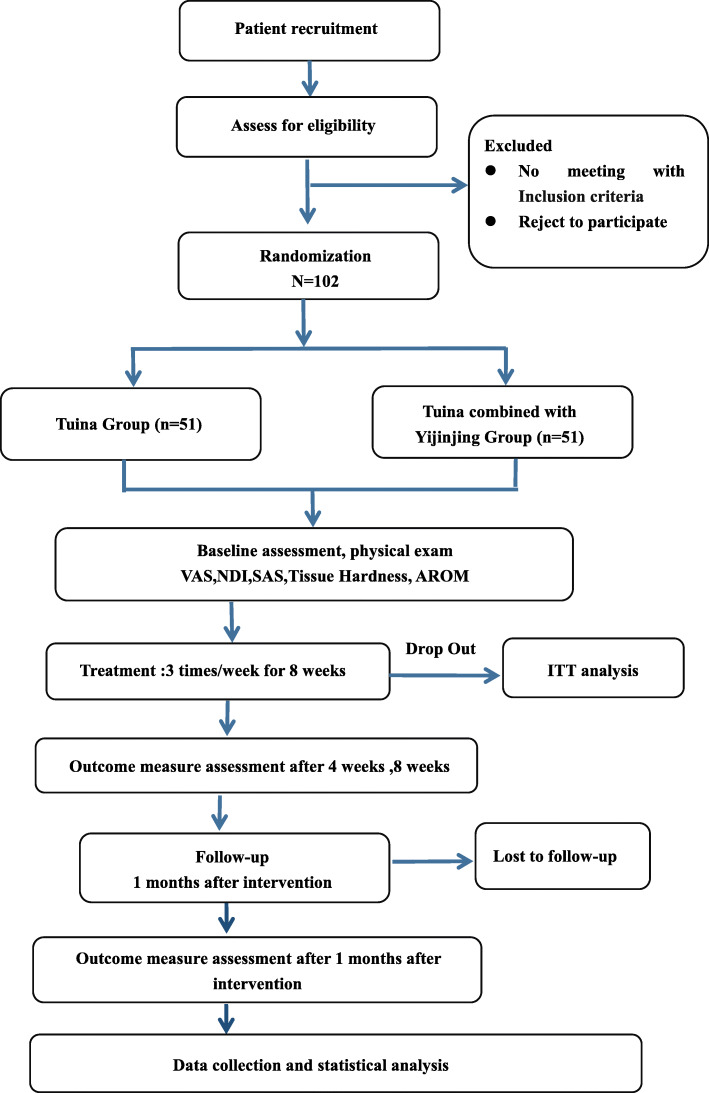
Trial flow chart. Diagram demonstrating the randomized controlled trial protocol. A total of 102 eligible NCNP patients will be randomly allocated to the group of Tuina and Tuina combined with Yijinjing in a 1:1 ratio. The dropout will be analyzed by ITT analysis

This present study is a single-center, randomized, and analyst-blinded controlled trial with two arms: Tuina group (control) and Yijinjng combined with Tuina group (intervention). The study protocol has been approved by the Regional Ethics Review Committee of Yueyang Hospital of Integrated Traditional Chinese and Western Medicine affiliated with Shanghai University of Traditional Chinese Medicine (project number: 2020-018). A total of 102 eligible NCNP patients will be recruited and assigned in a 1:1 ratio randomly. Written informed consent will be provided by all patients. Independent researchers who are blinded to the patient assignment will collect and analyze the outcome assessment and related data. The study design is illustrated in the flow chart in Fig. 1. The main treatment center will be the Shanghai University of Traditional Chinese Medicine.

### Participants and recruitment

Eligible participants include patients diagnosed with NCNP according to the base guideline for the chiropractic treatment of adults with neck pain which is summarized by GDC of Canada [37]. Patients with non-specific neck pain for more than 3 months (no relief for more than 2 weeks) for the first time in the Tuina Department of Yueyang Hospital of Integrated Traditional Chinese and Western Medicine affiliated with Shanghai University of Traditional Chinese Medicine will be informed about this trial. If the patient expresses interest in this trial, a clinical trial communicator will make a contract with him/her immediately and make a brief introduction about the trial. If the patient decides to take part in this trial, he/she will have a face-to-face interview in a reception room of the Shanghai University of Traditional Chinese Medicine. Patients who meet the inclusion criteria will join the trial after they sign the informed consent form.

We recruit the potential participants in the following ways:

1. Posters on affiliated hospitals of Shanghai University of Traditional Chinese Medicine and nearby community centers
2. On WeChat platforms and microblog
3. By printing ads on the newspaper

### Inclusion criteria

Participants who meet all the following criteria can be enrolled:

1. Aged 20–50
2. Individuals from either sex
3. Current neck pain (localized to the cervical or bilateral scapular region)
4. Negative sign of neck distraction test, Spurling’s neck compression test, and Adson’s test
5. Have neck pain symptoms of at least 3 months’ duration
6. Visual analog scale (VAS) ≥ 3 and Neck Disability Index (NDI) score ≥ 10 at recruitment time
7. No previous shoulder or neck surgery and no accompanying shoulder problems
8. Willingness to participate

### Exclusion criteria

Participants meeting any of the following criteria will be excluded from this trial:

1. Specific disorders of the cervical spine, such as disk prolapse, spinal stenosis, postoperative conditions, cervical radiculopathy, or myelopathy
2. History of whiplash injury and/or head/neck injuries
3. Are pregnant or have had a recent delivery
4. Response to prior treatment (a patient with neck pain radiating into the arm whose arm pain resolved with an injection or medication)
5. History of severe trauma, spasmodic torticollis, frequent migraine, fibromyalgia, shoulder diseases, inflammatory rheumatic diseases, tumor, osteoporosis, psychiatric illness, and obvious spinal deformity or neurological disease
6. No clinical treatment for neck pain in the past 3 months
7. Unable to speak or write Chinese in order to complete the questionnaires
8. Alcohol and drug abuse
9. Have an uncomfortable reaction to Tuina
10. Subjects with regular practice of Yijinjing, Qigong, or Yoga in the past 3 months
11. Poor cooperation

### Dropout criteria

Participants who do not complete the clinical protocol for the following reasons should be considered as dropped out:

1. The patient quits (poor efficacy or adverse reactions)
2. Loss to follow-up
3. Researchers remove the patient (poor compliance or serious adverse events)

### Comprehensive suspension criteria

The trial will be suspended if:

1. The investigators discover a significant safety problem.
2. The therapeutic effect is poor (we will assess the therapeutic effect at week 4).
3. There is a major mistake in the plan.
4. The sponsor has a huge problem in funding or management.

### Randomization and allocation concealment

The randomization list will be generated by a random number generator (Strategic Applications Software, version 9.1.3; SAS Institute Inc., Cary, NC, USA). The random numbers will be placed in an opaque envelope which has been numbered in order.

Before implementing random assignment, the research team will record the detailed information of each participant in the clinical center, including the new participant (name, date of birth, participant and center code, and date of inclusion) during reporting and preparation of a signed informed consent. The therapist will sequentially open the envelopes and allocate the participants accordingly. Eligible participants will be randomly assigned to the experimental group and the control group according to 1:1 equal proportion rules after the baseline assessment.

### Blinding

Patients will be informed of the type of treatment that they will receive. The therapists will know the allocation so they should learn how to communicate with patients to ensure treatment blinding. In order to reduce the risk of bias, evaluators, data managers, and statisticians will be unaware of the group assignments in the result evaluation procedures and data analysis. The blinding procedure will be operated until the data are locked.

### Interventions

The Tuina protocol used in this trial is the same as those used in our previous studies [38, 39]. It includes soft tissue manipulation and spinal manipulation, such as rolling, pressing, and tapping. Yijinjng for NCNP patients was designed on the basis of the textbook which has been used for teaching students in the universities of TCM [40, 41].

Participants in the Tuina group or the Yijinjng combined with Tuina group will receive Tuina treatment 3 times a week for 8 weeks. The treatment room will be controlled at 23–25° to ensure that the participants feel comfortable. The participants will be asked to rest for 15 min before Tuina treatment. They will be advised to lie in the prone position during the treatment. The intensity level of Tuina is based on physical examination and the therapist’s clinical experience, as well as after careful communication with each study participant. Tuina treatment will last for 25 min.

The participants who are in the Yijinjng combined with Tuina group will practice Yijinjing 3 times a week for 8 weeks. The patients will be assembled once a week for practicing Yijinjing together. The Yijinjing teacher will teach them how to practice Yijinjing. The teacher will also correct the wrong movement of patients. The patients will practice Yijnjing another two times per week at home. A digital video disk about the movements of Yijinjing in this trial will be provided to the participants. They can review the movements at home easily. The participants are asked to film themselves and sent it to the teacher by email or WeChat. The teacher will examine the participants’ video carefully and give the patients some advice about practicing Yijinjing. Yijinjing treatment will last for 30 min.

VAS, NDI, SAS, soft tissue hardness, and AROM will be assessed at the baseline and 4, 8, and 12 weeks.

### Tuina group

In this arm of study, the Tuina therapist will administer a three-step protocol intended to alleviate neck pain and restore neck function by relaxing the soft tissue of the neck and shoulder. The specific protocol used is described below.

#### Step 1: Soft tissue manipulation

Patients are instructed by the therapist to lie in the prone position and to relax their mind and body naturally. Non-specific chronic neck pain conditions will be carefully examined by postural and palpatory assessment prior to treatment. The therapist will relax soft tissue and stiff muscles of the neck and shoulder by pressing-kneading manipulation for 5 min. Then, the therapist will use his palms to roll the trapezius muscle gently so as to relax the back area for 5 min. The aim of this step is to resolve adhesion and increase general circulation.

#### Step 2: Clicking on the acupuncture point manipulation

The therapist will press and knead GB20, DU16, GB21, SJ14, and SI14 for 2 min each. This step is performed to unblock Qi stagnation and remove blood stasis by separating adherent fascicles. The amount of force used is determined by the patient’s Deqi sensation, often described as a dull pain, heaviness, numbness, or soreness, and it is commonly regarded as an indicator of manipulation effectiveness in acupuncture and Tuina [42, 43].

#### Step 3: Spinal manipulation

The spinal manipulation will be used after the above two steps have relieved the tensions of the muscles and soft tissues. The patient will be instructed to sit in an orthopnea position in order to ensure the safety of manipulation. First, the therapist can exert a gentle torque to align the patient’s neck area and use the shake method to relax this area. Then, the therapist will press one thumb on the deviated spinous process while another palm holds the lower jaw. Using gentle tractions and twisting of the neck, the therapist should hold this position for a moment and then made an abrupt pulling motion to advance the stretch by 5 to 10°.

### Yijinjing combined with Tuina group

**Fig. 2:**
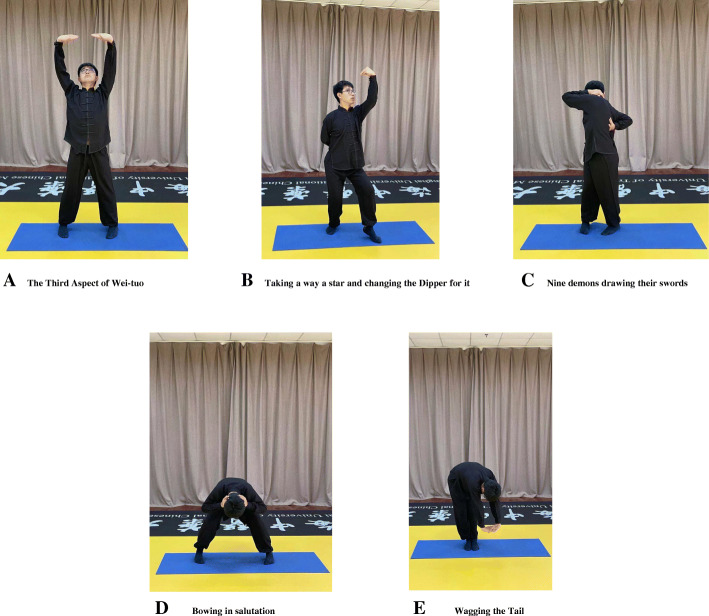
The main movements of Yinjining. The picture is from the “Teaching video of Yinjining for NCNP patients” self-recorded by the research group. The teacher in the video is Ziji Cheng, a member of the research group. **A** The third aspect of Wei-tuo. **B** Taking away a star and changing the Dipper for it. **C** Nine demons drawing their swords. **D** Bowing and salutation. **E** Wigging the tail

In this arm of study, the steps of Tuina treatment are the same as the Tuina group. The Yijinjing teacher will administer a five-step protocol intended to improve the therapeutic effects and adjust physical and mental conditions. The main movements of Yijinjing are shown in Fig. 2.

#### Step 1: The third aspect of Wei-tuo

Take a step to the left, raise the hands in front of the chest with palms up, fingertip facing. Rotate the wrists and raise the palms above the head. Slightly bend the elbow, look upward towards the dorsum of the palm. Lift the heels, stand on toes. Hold for 5 s. Then, make fists, rotate the wrists, and put the fists down to the waist slowly. Place the whole feet on the ground. Repeat the whole procedure 3 times in 6 min.

#### Step 2: Taking away a star and changing the Dipper for it

Move the right foot to the right front. Raise and straighten the right hand, direct the palms facing downward, slightly turn the head to the right side, and fix the eyes on the right palm. Bend the left elbow and put it naturally on the back of the body. Hold for 5 s. Then, put the hands to both sides of the body and relax when exhaling. Exchange the left side for exercising. Repeat the whole procedure 3 times in 6 min.

#### Step 3: Nine demons drawing their swords

Take a step to the left. Cross the hands over the chest and raise up. Separate the hands above the head. Put the left hand on the neck and the right hand to the back. Raise the head to the left 45° and twist waist. Pull the hands tightly when exhaling. Hold for 5 s. Then, put the hands to both sides of the body and relax when exhaling. Exchange to the right side for exercising. Repeat the whole procedure 3 times in 6 min.

#### Step 4: Bowing in salutation

Separate the legs and hold the head by the hands. Bend the waist between the knees. Stretch the head between the legs. Use finger to tap the head 7 times. Then, straighten the knee and waist when exhaling and stand upright. Repeat the whole procedure 3 times in 6 min.

#### Step 5: Wagging the tail

Take a step to the left. Cross the hands in front of the chest and raise the hands above the head. Fix the eyes to the hands. Then, bend the upper body upward and press down your hands to the ground. Raise up the head and open the eyes. Bend the body and place the heels when exhaling. Strengthen the body and lift the heels when inhaling. Repeat the whole procedure 3 times in 6 min. The patient can adjust the degree of flexion and extension according to his own physical condition.

### Study therapists

Tuina will be performed by a senior therapist who has studied Tuina and has held a practitioner’s license for more than 10 years. He/she must have received professional training in Tuina. Yijinjing will be conducted by a Yijinjing teacher with 10 years of teaching experience. Both Tuina therapist and Yijinjing teacher will be trained for a week. They must pass a test to ensure consistency of the study methods before participating in the trial.

### Allowance of concurrent treatment of patients

All other treatments for NCNP will be banned during the trial, including drug of non-drug. They may receive treatment which is not related to neck pain. Any change of concurrent treatments will be recorded.

### Outcome measurements

The efficacy between the two groups will be assessed by primary outcome measure: change in visual analog scale (VAS) four times (week 0, week 4, week 8, and week 12) (Table 1). The secondary outcomes measured at these four time points included (1) Neck Disability Index (NDI), (2) Self-Rating Anxiety Scale (SAS), (3) a digital pressure algometer used to assess tissue hardness, and (4) SH-105 used to assess cervical AROM (active range of motion).

**Table 1:**
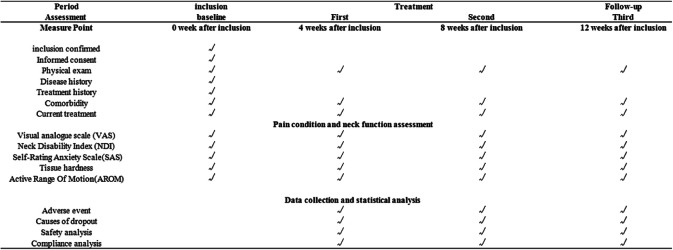
All measurements and measuring time points

### Primary outcome measurement

#### VAS

The intensity of NCNP will be measured by a scale of 10-cm horizontal line visual analog scale (VAS) [44]. The patients will be asked “How much pain do you have this moment?”, then the patient will mark on the 10-cm horizontal line visual analog scale. Zero means “absence of pain,” while 10 represent “the worst pain.” VAS has been proved as a valid and reliable outcome measure for recording pain with ICC = 0.96 to 0.98 according to a previous study [45].

### Secondary outcome measurements

#### NDI

The Neck Disability Index (NDI) will be used to measure the patients’ limitations in everyday life activities because of neck function [46]. It is most commonly used as a self-reported questionnaire in neck pain. It contains 10 questions, each of which comprises 6 potential answers ranging from 0 (no disability) to 5 (full disability), and the total of the NDI score varies between 0 and 50 points. The total NDI scores less than 4 indicate no disability; 5–14, mild disability; 15–24, moderate disability; 25–34, severe disability; and more than 35 points “complete disability.”

#### SAS

The anxiety level of NCNP patients will be measured by Zung Self-Rating Anxiety Scale (SAS) [47]. Twenty questions are divided into 4 groups: cognitive, autonomic, motor, and central nervous system symptoms. Each section is scored on four levels of anxiety intensity from “1” not at all to “4” very much and with a sum score between 20 and 80. A higher total score indicates a more severe anxiety level. The total raw scores range from 20 to 80. The raw score consequently needs to be converted to an “Anxiety Index.” The primary scores should be interpreted into the Anxiety Index. The clinical interpretation of one’s level of anxiety is as follows: 20–44, normal range; 45–59, mild to moderate; 60–74, marked to severe lever; and 75–80 extreme anxiety level.

#### Tissue hardness

Tissue hardness is measured by a digital algometer (OE-220, ITO, Tokyo, Japan) The measurement has been used to test the tissue hardness in previous study [48–50]. The measuring point is placed between C7 and acromion at the middle point of the upper trapezius muscle. The researcher puts the meter on the measuring point perpendicularly and push the force slowly. When hearing the deep sound, the researcher should stop pushing and read the number. To standardize the speed of using this application, the researchers responsible for this measurement will practice 1 week before the study. They must explain the measurement by demonstrating at the thenar region of the hand. Three soft tissue measurements will be preformed at each point with an interval of 30 s between the two measurements; the mean of three measurements will be recorded.

#### Active range of motion (AROM)

Cervical active range of motion is measure by Spain ScanTM SH-105 (Ad-Or Medical Technologies Ltd., Israel). The validity and reproducibility of this measurement have been proved by many researchers [51, 52]. SH-105 is composed of a goniometer and a computer. The data will be transmitted by Bluetooth. The patient will be seated with a straight back leaning against the back of a chair. The goniometer will be placed on the center of the cranial. The researcher switches the mode and long press the “start/end” button. When hearing the prompt sounds, the patient will be asked to flex forward to the limit. The patient will be instructed to stop at the point where pain symptoms preform. The research press the “start/end” button again, and the computer will automatically record the data. Each movement (flexion-extension, lateral flexion as well as rotation) will be measured in this way three times. The mean of each movement will be recorded.

### Safety evaluation

The safety of patients will be monitored in every visit. Two attending physicians and two responsible spine care specialists will be responsible for the collaboration and guidance of clinicians during each clinical trial and implementation. The two chief spinal surgeons constitute the endpoints Adjudication Committee, which is responsible for the overall supervision of the clinical trials. The AES in trial are changes in pain, syncope, vertigo, and disability. For any AES, no matter if it is or not caused by intervention, the treatment will be stopped immediately. The patient should take any medical aids to alleviate symptoms. The adverse events should be reported to the relevant responsible unit and the ethics committee in time. If the adverse events are confirmed to be related to the study, the regional Ethics Review Committee of Yueyang Integrated Traditional Chinese and Western Medicine Hospital affiliated to Shanghai University of Traditional Chinese Medicine has the right to suspend the study. If an adverse event occurs during the clinical trial, medical experts will be engaged to assess and investigate the actual cause, and medical and financial compensation will be made to participants.

### Follow-up

To evaluate the long-term efficacy and safety of the intervention, we will follow up the patients after treatments for 1 month. During this period, patients would not receive any treatment. No researcher will supervise them. At the end of the week, the outcome assessor will make a contract with patients by WeChat, telephone, e-mail, etc. They will be asked to come to the reception room and fill in the relevant scales above. Tissue hardness and AROM will be measured at the same time.

### Sample size calculation

The following two hypotheses are related to the differences between the two groups.

- H0:μ1 − μ2 ≤ Δ
- H1:μ1 − μ2 > Δ

where μ1 is the VAS score for treating 8 weeks in the Tuina group, and μ2 is the VAS score for treating 8 weeks in the Tuina combined with Yijinjing group.

According to a previous clinical study in China [53], the mean and standard deviation of VAS in the Tuina group (*n* = 34) after the intervention was 5.5 and 1.1, respectively. The mean and standard deviation of VAS in the Tuina combined with neck exercise group (*n* = 34) after the intervention was 4.7 and 1.3, respectively.

The following formula was used to calculate the sample size in this trial:

$$ n=\frac{2{\left({z}_{\alpha /2}+{z}_{\beta}\right)}^2\times \kern0.5em {\sigma}^2}{{\left({\mu}_2-{\mu}_1-\varDelta \right)}^2} $$

$$ {\displaystyle \begin{array}{l}n=\frac{2{\left(1.96+0.84\right)}^2\times {1.3}^2}{{\left(4.7-5.5-0\right)}^2}=42\\ {}\end{array}} $$

(*α* = 0.05 *β* = 0.2, superiority design, the two-sided test, *Δ* = 0)

Considering a dropout rate of 20%, each group will require 51 cases. Therefore, a total of 102 participants should be recruited for this randomized controlled trial (RCT).

### Statistical analysis

All statistical analyses will be performed with the SPSS software (SPSS, version 24.0, SPSS Inc., Chicago, IL, USA) by statisticians who are independent of the research team and blinded to the group allocation. Data analysis will be based on the intention-to-treat (ITT) principles. The statistical significance was accepted for values of *p* < 0.05. Participants who fail to complete the study will be treated as having no change from baseline at all times. Descriptive statistics will be used to compare demographic and baseline information and evaluate the credibility of the groups. The normality of data will be tested by the Kolmogorov-Smirnov test. Parametric statistics (Tukey test) or non-parametric statistics (Wilcoxon rank sum test) will be used for the within- and between-group according to the results of homogeneity and normality analysis. If the data does not conform to a normal distribution, a covariance analysis will be used. The efficacy will be measured at four time points. A repeated measures analysis of variance will be conducted to analyze dependent variables (from baseline and follow-up). The Bonferroni and Dunn tests will be used for multiple comparisons. The intra-group comparison (comparison between baseline and follow-up) will be tested by a two-sided paired *t* test. The difference of categorical variables (VAS, NDI, SAS, PPT, AROM) and adverse effects between the groups will be analyzed by using The chi-square test or Fisher’s exact test. All numerical data will be presented as the mean ± SD, and categorical variables will be described with percentages (%). If it is necessary, post hoc analyses will be performed. The confidence interval will be established at 95% and the significance level at 0.05.

### Data collection and management

The outcome assessors will use the CRFs to collect the data which consists of a questionnaire-based assessment of treatment effects, active range of motion (AROM), tissue hardness, adverse events, and safety evaluations. Then, two data administrators who do not belong to the research team and blinded to group allocation will independently receive the completed CRF and enter it into the Excel database (Microsoft, Redmond, WA, USA). They must complete rigorous data monitoring training. Then, they enter the real-time data into the China Clinical Trial Registry (http://www.chictr.org.cn), where the electronic data management system will be used for real-time tracking and monitoring of test data from the Science and Technology Department of Yueyang Hospital.

### Quality control

Quality control will be conducted during the study. The head of research center will be responsible for the coordination, development, and quality control of all the programs in the trial. During the trial, the reviewers who are independent of the researcher and sponsor will extract 10% of the case reports from the CRFs to check the data every 3 months, including logic problems, and test value determination, abnormal safety indicators after treatment, vacancy values, compliance nature, normative, completeness, consistency, etc. All research members in this study will receive professional training before the study. Any corrections or revisions of the protocol will be discussed by the ethics committee of Yueyang Hospital of Integrated Traditional Chinese and Western Medicine. Detailed information of changes will be kept.

### Ethical considerations

The study collects data from patients with NCNP. Informed consent is required from the patients. Patients can terminate participation at any time.

The results of the trial will be shown in tables and figures only, and no individual will be identified. All data collected from this study can only be used for this research. All members of the research team have ethical principles of confidentiality.

In addition, we will try our best to deal with ethical issues which arise during the study. We estimate that the benefits of the study far outweigh any possible risks. The trial has been approved by the ethics committee of Yueyang Hospital of Integrated Traditional Chinese and Western Medicine, which is affiliated with Shanghai University of Traditional Chinese Medicine (2020-018) and registered on ChiCTR (2000036805).

## Discussion

Non-specific chronic neck pain (NCNP) is a common and high prevalence musculoskeletal problem in the world. It can cause ADL, work disability and economic cost, and psychological stress [54, 55]. Because of the high recurrence rate, a scientific and reasonable intervention should be explored and promoted. It can not only relieve symptoms and reduce the burden of individuals and the society, but also decrease the complication and improve the quality of life. Yijinjing and Tuina are important components of traditional Chinese medicine. They have been used for thousands of years to keep people healthy. Quite a few people know the concrete operating procedure, advantages, and efficacy of such interventions. Therefore, the aim of this study is to investigate whether Yijinjing combined with Tuina has any superiority to Tuina.

According to TCM, a healthy human body depends on the coordination of the internal organs and the harmony of qi and blood. The main reason of pain will be attributed to qi stagnation and blood stasis. In this study, relaxing manipulation, clicking on the acupuncture point manipulation, and neck structural rectification are chosen to release adhesion and smooth joint movement so as to alleviate pain and improve joint movement condition. By using these manipulations, the circulation of qi and blood will be promoted. Five movements of Yijnjing which are specifically for the neck are chosen. These movements include neck flexion, rotation, and lateral flexion. The features of Yijijing are the harmony of body, breath, and mind. It puts emphasis on the unity of strength and meditation by using static postures and dynamic movements. It can circulate qi and blood, strengthen the muscles and nourish the tissues and organs, and also arrest spasm [56]. The previous study demonstrated that regular and long-term training of Yijinjing can raise the skeletal muscle strength and improve motor function and ADL [57, 58]. A clinical research reported that after 6 months, Yijinjing multiple factors of depression and anxiety dropped significantly. It indicated that Yijinjing can improve patients’ mental conditions [59].

The present trial is a comparative efficacy of Tuina (control) and Yijinjing combined with Tuina (intervention) for the physical and mental symptoms of NCNP patients. We want to explore whether Yijinjing combined with Tuina is better than Tuina. We will evaluate three aspects of neck pain: pain, physical function, and mental function. we will use validated scales and questionnaires and some measuring instruments to assess the clinical outcomes.

Pain is the most important symptom of neck pain, so visual analog scale (visual analog scale) will be used as the primary outcome. It can evaluate the intensity of neck pain. One of the secondary outcomes is NDI, which can evaluate the limitation of daily life because of NCNP. Cervical active range of motion (AROM) which can evaluate the neck active range of motion is measured by using an easy equipment. SAS can evaluate the anxiety level of patients. Tissue hardness will be measured by a tissue hardness meter and expressed in numbers.

To the best of our knowledge, no study has proved the efficacy of Yijinjing combined with Tuina for patients with NCNP.

High-quality clinical data will be collected because of the rigorous experimental design. The efficacy of this specific intervention protocol for treating NCNP will be evaluated by these data. We hope that this study will provide a solid foundation for the treatment of NCNP, as well as Tuina and Yijinjing research.

### Strengths and limitation of the study

The strength of this trail includes the randomized study design, the pre-defined sample size, and the application of Yijinjing which is designed by an experienced expert. Secondly, the use of standardized measurement instruments will become very important inclusions for the study. Finally, many resistance trainings are limited to equipment-based ones in rehabilitation organizations or hospitals. They cost high and fail to combined positive and passive movement. As a kind of traditional Chinese exercise, it is easy for people to master in a short time. Moreover, Yijinjing does not require expensive equipment. It can not only strengthen the bones and sinews, but also nourish organs. It will make a positive effect on physiological structures and pathological conditions.

There are several limitations to this study. The first is the blinding of the therapist and patients. The nature of the interventions makes it impossible to blind them during the study. Secondly, self-reported results, such as the VAS and SAS, also have limitations because they may be affected by the placebo effect and expected results. Finally, there is in fact no control group. We use Tuina therapy as a control which is considered experimental.

### Trial status

This trial is recruiting patients now. Participant recruitment started in May 2020 and is expected to end in December 2021.

## Data Availability

The authors will submit the research results to a peer-reviewed journal. The data will be put into the China Clinical Trial Registry (http://www.chictr.org.cn). Data and materials can be obtained from the corresponding author after the trial.

## Abbreviations

NCNP: Non-specific chronic neck pain
VAS: Visual analog scale
NDI: Neck Disability Index
SAS: Self-Rating Anxiety Scale
AROM: Active Range of Motion
ROM: Range of motion
EMG: Electromyography
ADL: Activities of daily living
CRF: Case report form
